# Synthesis and crystal structure of poly[μ-2,6-di­methyl­pyrazine-μ_3_-iodido-μ_2_-iodido-dicopper(I)] with an unusual CuI substructure

**DOI:** 10.1107/S2056989026007358

**Published:** 2026-07-23

**Authors:** Christian Näther

**Affiliations:** a Institut für Anorganische Chemie, Universität Kiel, Max-Eyth.-Str. 2, 24118 Kiel, Germany; University of Aberdeen, United Kingdom

**Keywords:** crystal structure, coordination polymer, copper(I) iodide, 2,6-di­methyl­pyrazine

## Abstract

The asymmetric unit of the title compound, [Cu_2_I_2_(C_6_H_8_N_2_)]_*n*_, consists of six crystallographically independent Cu^I^ cations, six unique iodide anions and three independent 2,6-di­methyl­pyrazine ligands, all of them located in general positions. Three of the six copper cations display trigonal planar coordinations (one 2,6-di­methyl­pyrazine ligand and two iodide ions), whereas the remaining cations are tetra­hedrally coordinated (one 2,6-di­methyl­pyrazine ligand and three iodide ions). In the extended structure, two different CuI substructures are observed. In one of them, discrete (CuI)_4_ units are observed, which show a ladder-like arrangement. Two of the cations are threefold, the other two cations are fourfold coordinated and are linked by two μ-1,1 and two μ-1,1,1 bridging iodide anions. In the other substructure (CuI)_2_ rings built up of one threefold and one fourfold coordinated copper cation as well as one μ-1,1 and one μ-1,1,1 bridging iodide anion are connected into chains. These CuI substructures are linked by bridging 2,6-di­methyl­pyrazine ligands into layers that lie parallel to the *ab* plane. The layers are connected by a number of C—H⋯I inter­actions. Analyzing the CuI substructures in related copper(I) iodide coordination compounds with pyrazine deriv­atives as neutral ligands revealed that the same ladder-like CuI substructure is observed in all of them, which is completely different from that observed in the title compound.

## Abstract 2

In the crystal structure of the title compound, two different CuI substructures are observed, which condense into layers by way of bridging 2,6-di­methyl­pyrazine ligands.

## 1. Chemical context

Coordination compounds based on copper(I) halides with chloride, bromide and iodide anions are characterized by a variety of Cu*X* substructures in which the copper cations are linked by bridging halide anions into dinuclear units, single or double chains, and numerous examples of such compounds are reported in the literature (Kromp & Sheldrick, 1999 ▸; Li *et al.*, 2005 ▸; Peng *et al.*, 2010 ▸). The structural variability of the CuI networks might also be one reason why many isomeric or polymorphic networks are observed (Näther & Jess, 2003 ▸; Park *et al.*, 2012 ▸; Peng *et al.*, 2010 ▸; Näther *et al.*, 2003 ▸). These substructures can further be connected into more condensed networks if bridging neutral coligands such as, for example, pyrazine or 4,4′-bi­pyridine derivatives are used (Näther & Jess, 2001 ▸; Näther *et al.*, 2001 ▸, 2002 ▸). The actual Cu*X* substructure (*X* = Cl, Br, I) predominantly depends on the ratio between the copper(I) halide and the neutral coligand and to some extent on the coordination behavior of the coligand, whether it acts as terminal or bridging ligand.

The crystal structure of CuI(C_4_H_4_N_2_) (C_4_H_4_N_2_ = pyrazine) for example is built up of copper cations that are linked by μ-1,1 bridging iodide anions into single chains, which are further connected by bridging pyrazine ligands into layers (Cambridge Structural Database refcodes HAWWIO and HAWWIO01; Malastean *et al.*, 2017 ▸). For this composition, however, a second isomer exists in which (CuI)_2_ rings are linked by bridging pyrazine ligands into layers (HAWWIO02; Cappucino *et al.*, 2019 ▸). There is also a pyrazine-deficient compound with the composition (CuI)_2_(C_4_H_4_N_2_) in which CuI double chains are observed, that are linked into layers by the pyrazine ligand (AGIYEU, Groeneman & Atwood, 2001 ▸; AGIYEU01, Blake *et al.*, 1999 ▸; AGIYEU02, Goforth *et al.*, 2003 ▸). This shows that with increasing ratio between the copper(I) halide and the coligands, more condensed Cu*X* networks are obtained.

However, CuI double chains are also observed in CuI(pyridine) (CUIPYS, Eitel *et al.*, 1980 ▸), even if the ratio between CuI and the coligand is only 1:1, which can be traced back to the fact that this coligand cannot act as bridging ligand and therefore, even for this stoichiometry, a more condensed network with double chains is observed. A similar structure is also observed in CuI(2,6-di­methyl­pyrazine) (TONQOE; Kitada & Ishida, 2014 ▸ and TONQOE01; Zhang *et al.*, 2014 ▸), because in this ligand one of the two N atoms is shielded by the two neighboring methyl groups, which makes metal coordination to this N atom much more difficult. However, this ligand can also act as bridging ligand, as is the case in (CuCl)_2_(2,6-di­methyl­pyrazine), which is already reported in the literature (YEFPOR; Fan *et al.*, 2015*a* ▸). As expected, in this compound CuCl double chains are found that are connected into layers by bridging 2,6-di­methyl­pyrazine ligands. Therefore, one might expect that even with copper(I) iodide, a 2,6-di­methyl­pyrazine-deficient compound with the composition (CuI)_2_(2,6-di­methyl­pyrazine) might be accessible, which should show a CuI substructure similar to that in (CuI)_2_(pyrazine) or CuI(pyridine). To verify this assumption, CuI was reacted with 2,6-di­methyl­pyrazine in aceto­nitrile, which leads to the formation of crystals of the title compound (CuI)_2_(C_6_H_8_N_2_) (C_6_H_8_N_2_ = 2,6-di­methyl­pyrazine) (**I**), which were characterized by single crystal X-ray diffraction.
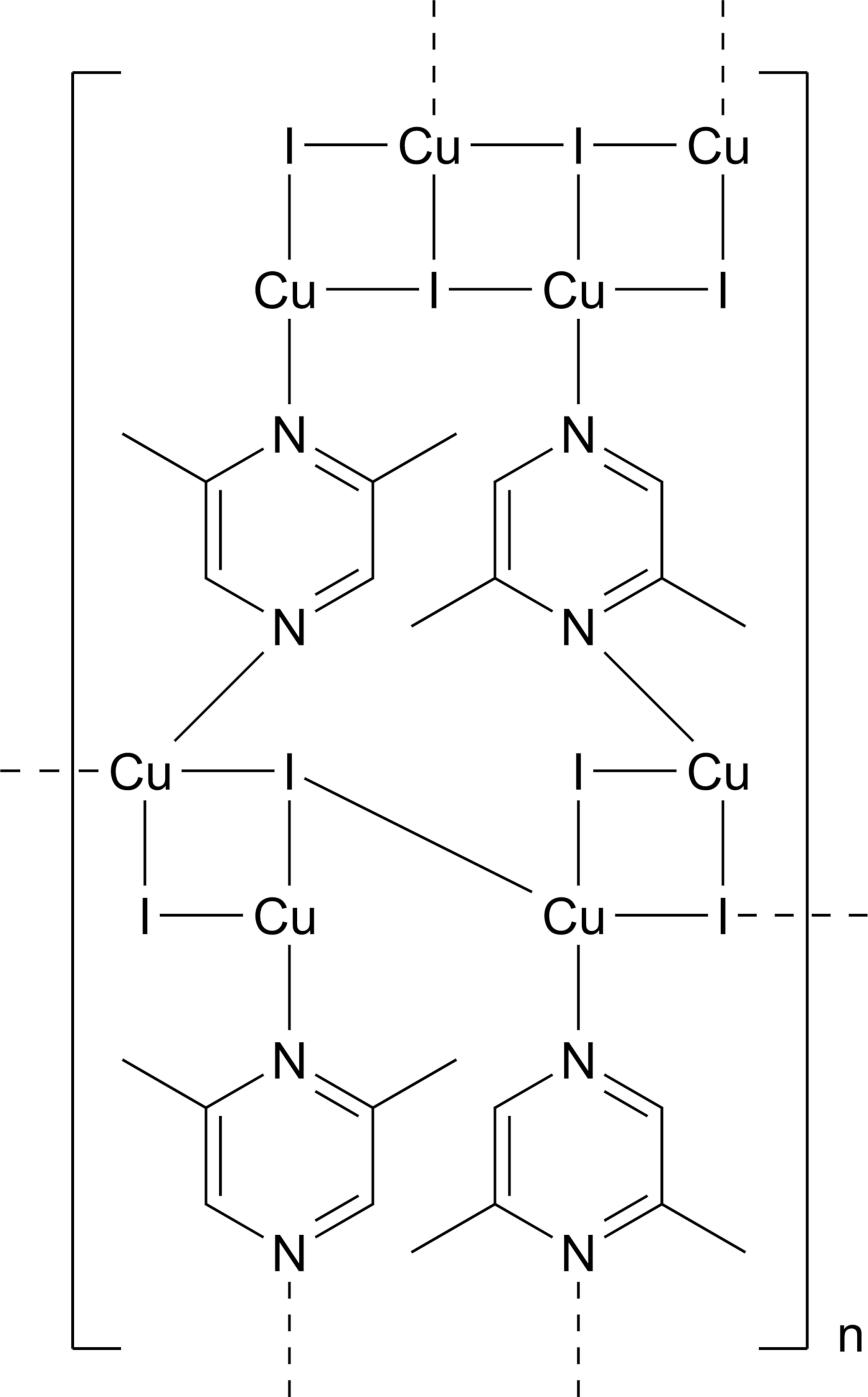


## 2. Structural commentary

**Figure 1:**
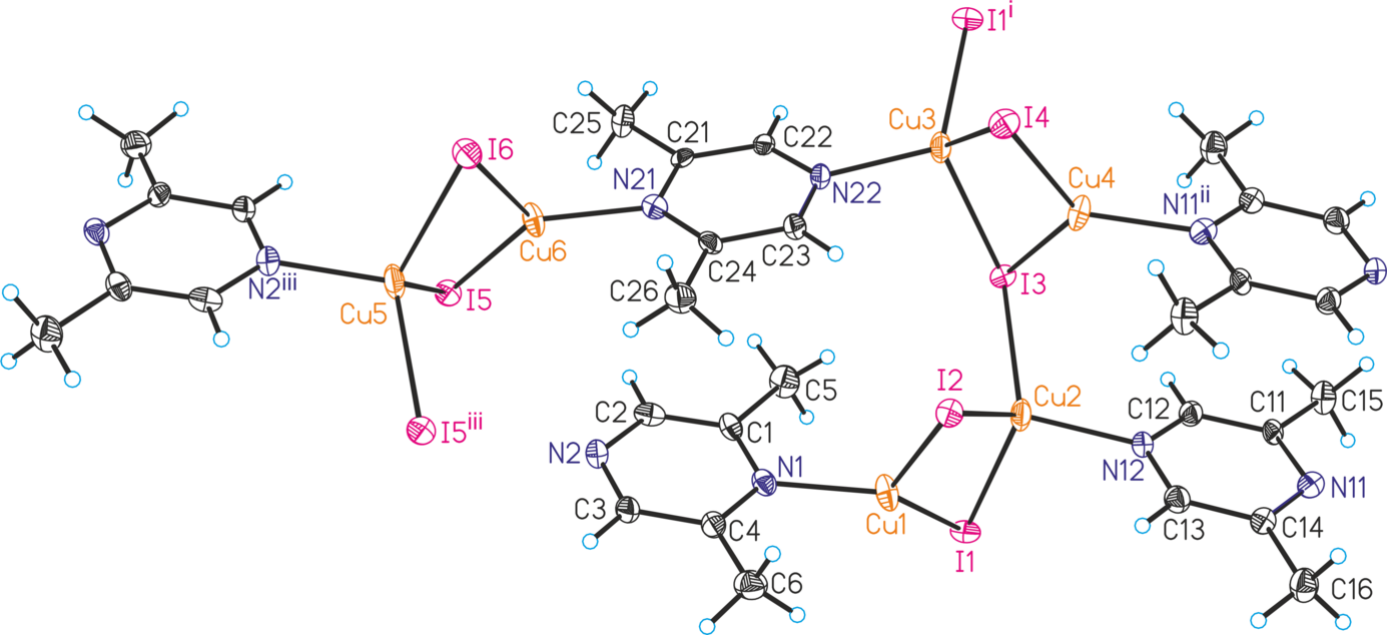
The asymmetric unit of (**I**) expanded to show the complete copper coordinations with labeling of selected atoms and displacement ellipsoids drawn at the 50% probability level. Symmetry codes: (i) *x* − 1, *y*, *z*; (ii) −*x* + 1, −*y* + 1, −*z* + 1; (iii) −*x* + 1, −*y*, −*z* + 1.

The asymmetric unit of (**I**) is built up from six crystallographically independent copper(I) cations and iodide anions as well as three crystallographically independent 2,6-di­methyl­pyrazine ligands, with all atoms located in general positions (Fig. 1 ▸) in space group *P*2_1_/*c*. Three of the copper cations (Cu1, Cu4 and Cu6) are threefold coordinated by two iodide anions and one 2,6-di­methyl­pyrazine ligand (Table 1 ▸). The sum of angles for these Cu^I^ cations amount to 119.1 (2), 120.0 (2) and 120.0 (2) °, respectively, which shows that they are in a trigonal–planar coordination (Table 1 ▸). Moreover, between the threefold and fourfold coordinated Cu^I^ cations relatively short Cu⋯Cu distances of 2.5645 (12), 2.5876 (11) and 2.5746 (11) Å are observed, indicating *d*^10^–*d*^10^ inter­actions (Jansen, 1987 ▸). In contrast, Cu2, Cu3 and Cu5 are fourfold coordinated by three iodide anions and one 2,6-di­methyl­pyrazine ligand (Fig. 1 ▸) and from the bond angles it is obvious that they show a distorted tetra­hedral coordination (Table 1 ▸).

**Figure 2:**
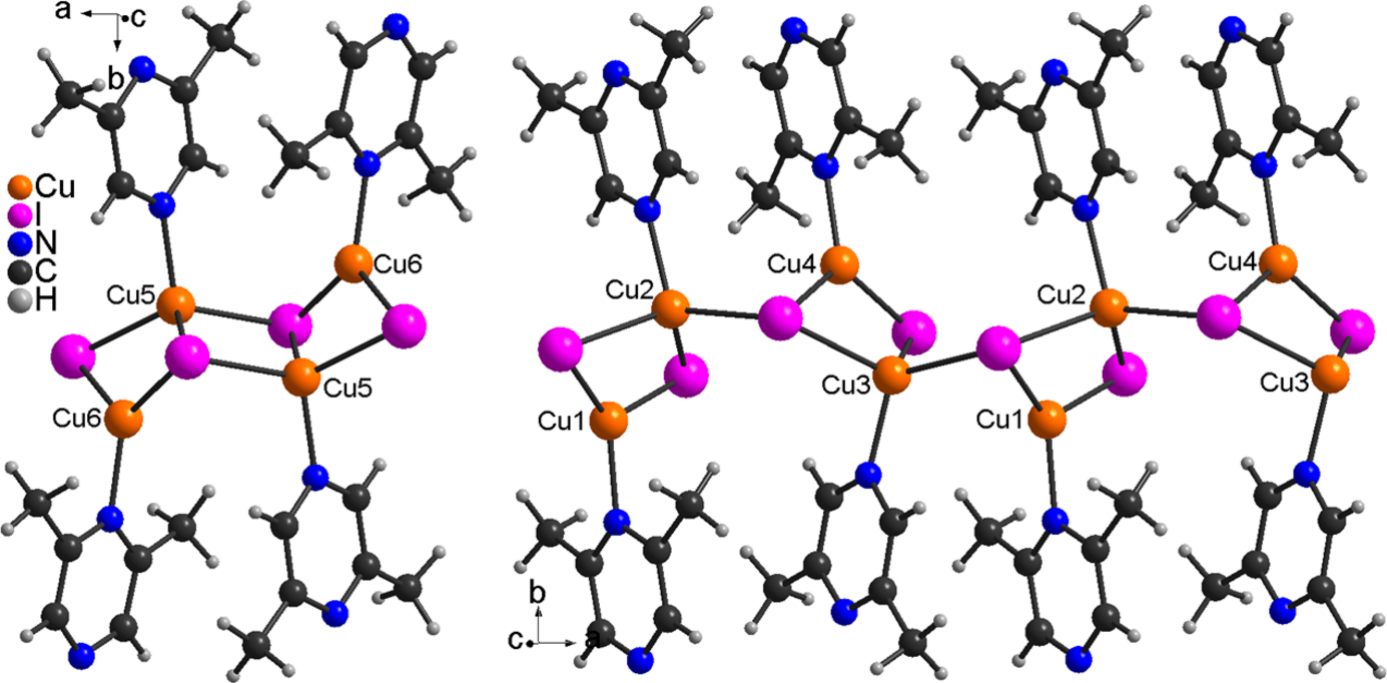
Details of the extended structure of (**I**) with views of the discrete (CuI)_4_ units (left) and the CuI chains (right).

**Figure 3:**
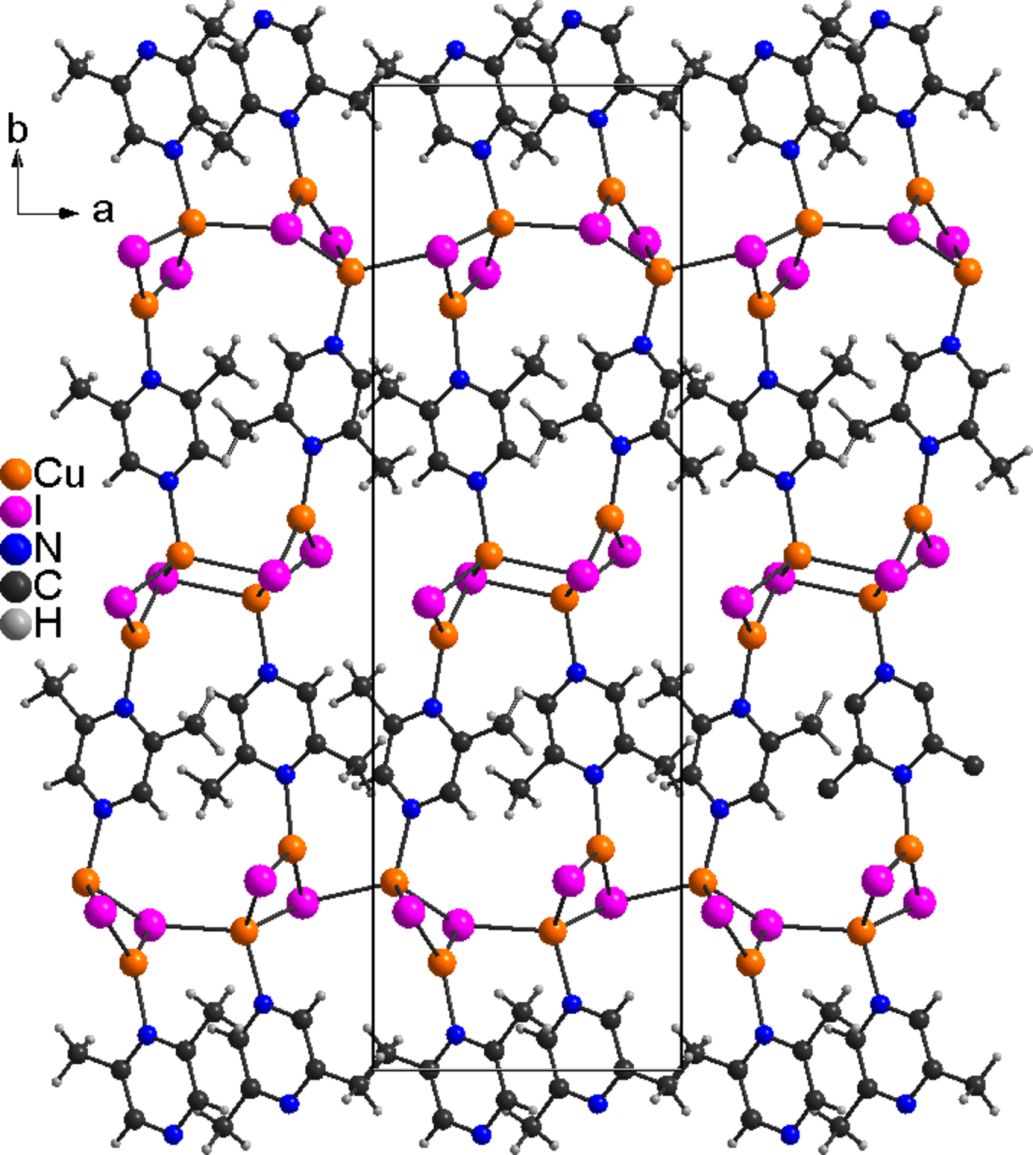
The crystal structure of (**I**) in a view along the crystallographic *c*-axis direction.

The copper cations are linked by the iodide anions into two different CuI substructures (Fig. 2 ▸). In one of them discrete ladder-like (CuI)_4_ units are found, which consist of three condensed four-membered rings, in which each copper(I) cation is coordinated to one μ-1,1 and one μ-1,1,1 bridging iodide anion (Fig. 2 ▸: left). The second CuI substructure consists of four-membered CuI rings in which the threefold coordinated copper(I) cations are linked to two μ-1,1 bridging iodide anions, whereas the second cation is connected to one μ-1,1 and one μ-1,1,1 bridging iodide anion (Fig. 2 ▸: right). These four-membered rings are connected into chains by the μ-1,1,1 bridging iodide anions (Fig. 2 ▸: right). The two different CuI substructures are linked into layers that lie parallel to the *ab* plane by bridging 2,6-di­methyl­pyrazine ligands (Fig. 3 ▸). The two different substructures alternate along the crystallographic *b*-axis direction, leading to the long unit-cell axis (Fig. 3 ▸).

**Figure 4:**
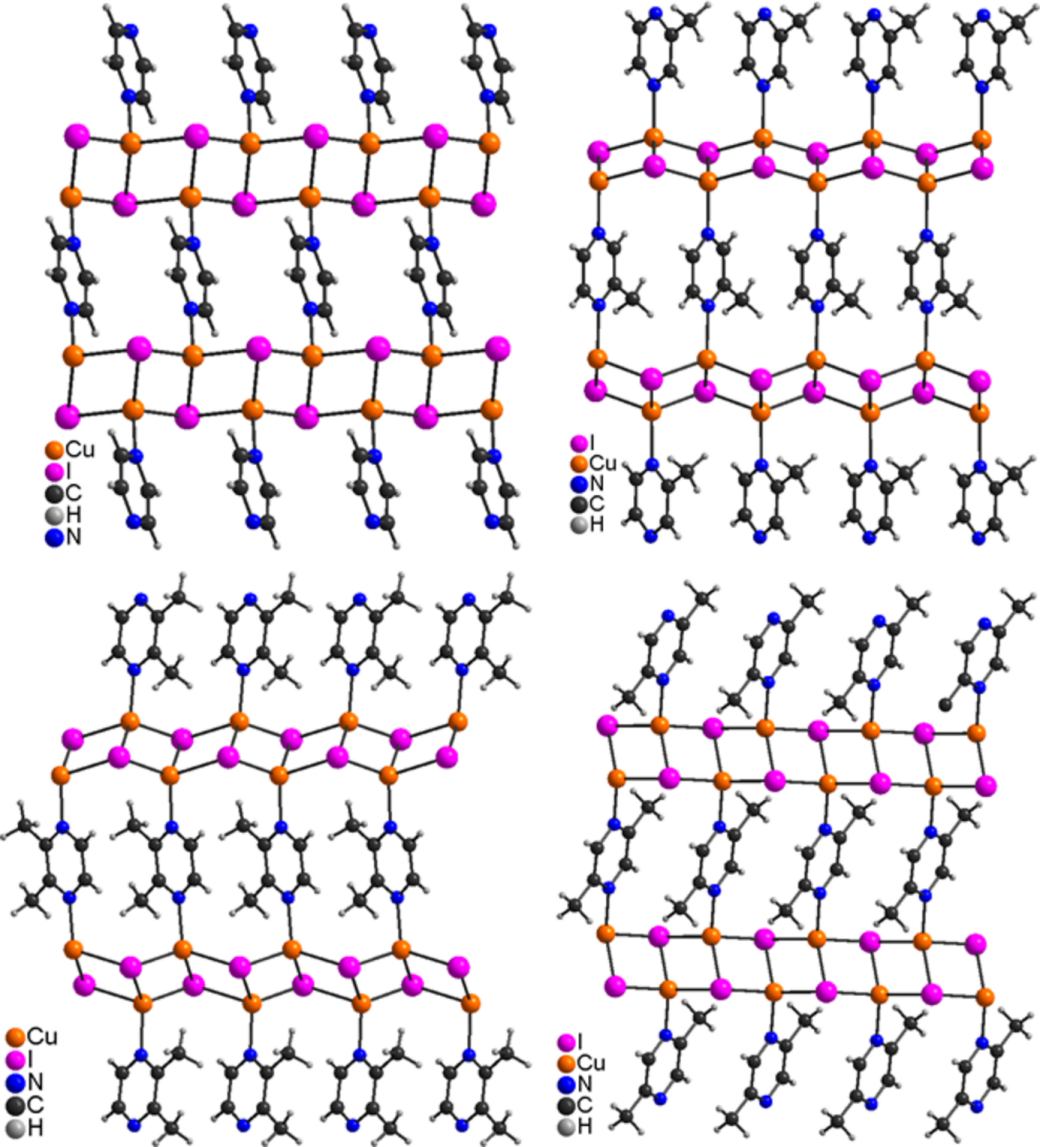
Crystal structure of CuI coordination polymers of the general composition CuI(*L*) with *L* = pyrazine (top left), 2-methyl­pyrazine (top right), 2,3-di­methyl­pyrazine (bottom left) and 2,5-di­methyl­pyrazine (bottom right), retrieved from the literature.

At first glance, this CuI substructure seems to be very unusual and therefore the crystal structures of other CuI compounds with similar coligands were analyzed. Compounds with the same ratio between CuI and the coligand and that consists of pyrazine derivatives with methyl groups are initially suitable for this purpose. The crystal structure of (CuI)_2_(pyrazine) has already been mentioned in the *Chemical context* section (AGIYEU; Groeneman & Atwood, 2001 ▸, AGIYEU01; Blake *et al.*, 1999 ▸ and AGIYEU02; Goforth *et al.*, 2003 ▸). It consists of ladder-like CuI double chains in which the copper cations are tetra­hedrally coordinated by one N atom of the pyrazine ligand and three μ-1,1,1 bridging iodide anions and are linked by the bridging pyrazine ligands into layers (Fig. 4 ▸: top left). The corresponding compound with methyl­pyrazine is also known (XEBMUM; Rossenbeck & Sheldrick, 2000 ▸) and it shows the same CuI substructure, which is connected into layers by the methyl­pyrazine coligands (Fig. 4 ▸: top right). For di­methyl­pyrazine derivatives two more isomers exist, and for both of them corresponding CuI compounds are reported. These include (CuI)_2_(2,3-di­methyl­pyrazine (LIDYAZ; Jess *et al.*, 2007 ▸ and LIDYAZ01, Xu *et al.*, 2020 ▸) and (CuI)2(2,5-di­methyl­pyrazine) (MUHQOV; Näther *et al.*, 2002 ▸ and MUHQOV01; Zhang *et al.*, 2014 ▸). Both of these compounds show the same structure as the pyrazine and methyl­pyrazine CuI compounds (Fig. 4 ▸: bottom).

**Figure 5:**
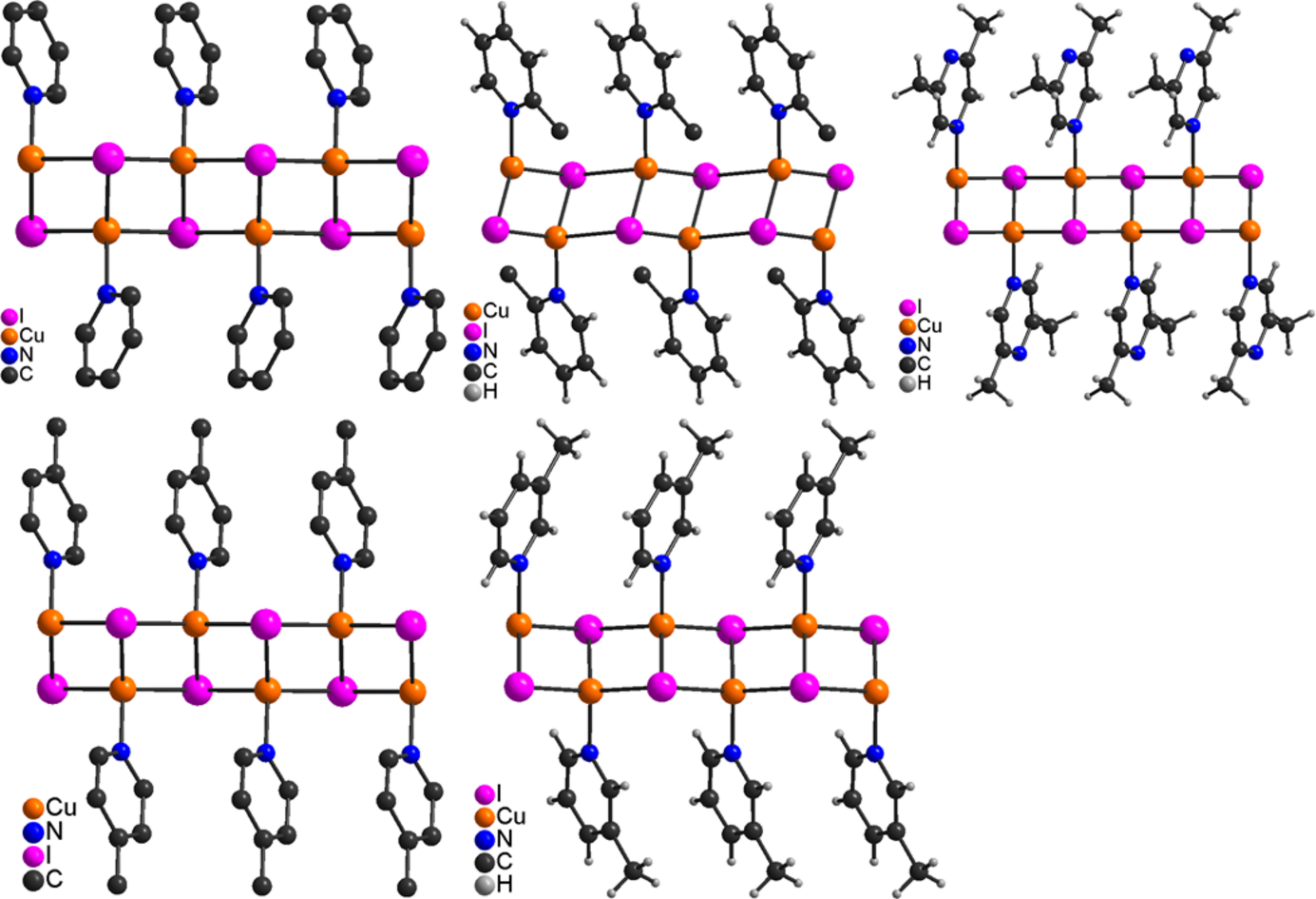
Crystal structure of CuI coordination compounds with the general composition (CuI)_2_(*L*) with *L* = pyridine (top left), 2-methyl­pyridine (top middle), 2,6-di­methyl­pyrazine (top right), 4-methyl­pyridine (bottom left) and 3-methyl­pyridine (bottom right), retrieved from the literature. Note that for two structures no H-atom coordinates were given.

Similar CuI networks can also be expected for compounds with the general composition CuI(*L*) if the coligand can act only as terminal ligand. This is the case, for example, in pyridine and its methyl derivatives. Consequently, CuI double chains are also observed in CuI(pyridine) (CUIPYS; Eitel *et al.*, 1980 ▸), which was also mentioned in the *Chemical context* section (Fig. 5 ▸: top left). For methyl­pyridine, three isomers exist and with all of them corresponding CuI compounds are known. These include CuI(2-methyl­pyridine) (FALYEW; Rath *et al.*, 1986 ▸), CuI(3-methyl­pyridine) (MICMUG; Cariati *et al.*, 2000 ▸) and CuI(4-methyl­pyridine) (MICNAN; Cariati *et al.*, 2000 ▸) and in all of them the same CuI double chains are found (Fig. 5 ▸). Finally, CuI double chains are also observed in CuI(2,6-di­methyl­pyrazine) (MUHQOV; Näther *et al.*, 2002 ▸ and MUHQOV01, Zhang *et al.*, 2014 ▸), where a bridging coordination is more difficult because of the two neighboring methyl groups.

Summarizing, the analysis of related compounds indicates that the CuI substructure with ladder-like CuI double chains seems to be very stable and therefore, the question arises of why the CuI substructure in the title compound is completely different. In this context it is mentioned that in (CuCl)_2_(2,6-di­methyl­pyrazine) the expected layered structure with CuCl double chains is observed (YEFPOR; Fan *et al.*, 2015*a* ▸). What is common for all compounds with Cu*X* double chains discussed here is the fact that the copper(I) cations are always tetra­hedrally coordinated. In contrast, in the title compound only half of the cations are tetra­hedrally coordinated whereas the other half are in a trigonal–planar arrangement. Inter­estingly, all threefold-coordinated copper(I) cations coordinate to the N atoms of the 2,6-di­methyl­pyrazine ligand that is shielded by the two neighboring methyl groups. This indicates that, in contrast to (CuCl)_2_(2,6-di­methyl­pyrazine), the copper cation in the title compound paired with much larger iodide anions can only effectively coordinate to the N atom that is adjacent to the two methyl groups if the cation is in a threefold coordination.

## 3. Supra­molecular features

**Figure 6:**
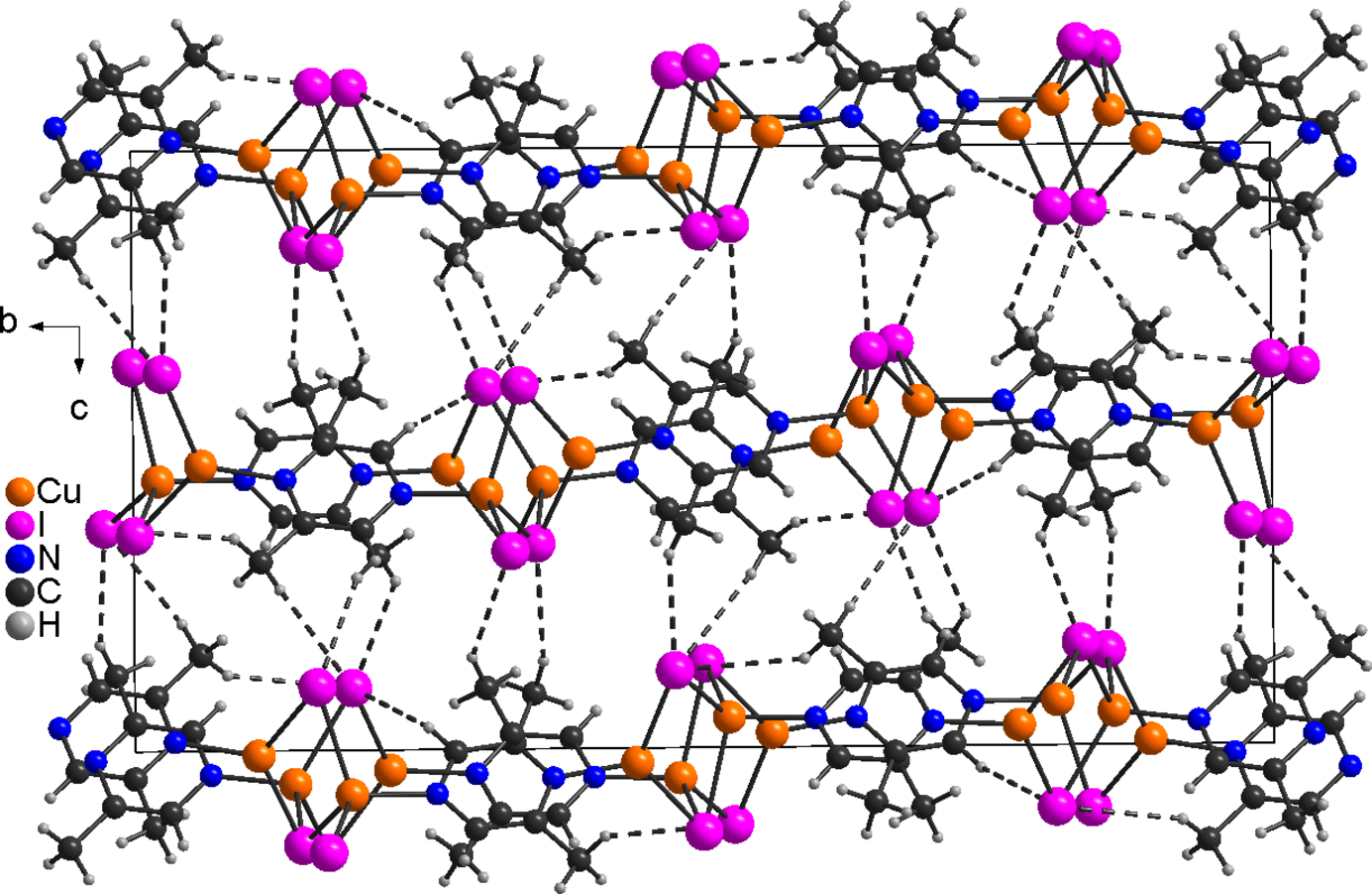
Crystal structure of (**I**) in a view along the crystallographic *a*-axis direction with C—H⋯I inter­actions shown as dashed lines.

In the extended structure of (**I**), the layers are stacked perpendicular to the crystallographic *a* axis (Fig. 6 ▸). A large number of C—H⋯I contacts are observed between the layers, several of which have C—H⋯I angles that are close to linear, indicating that these are significant inter­actions (Table 2 ▸).

## 4. Database survey

A search in the Cambridge Structural Database (CSD Version 5.43, 2025; Groom *et al.*, 2016 ▸) using CONQUEST (Bruno *et al.*, 2002 ▸) revealed that only two copper(I) halide compounds with 2,6-di­methyl­pyrazine are reported, *viz*. CuI(2,6-di­methyl­pyrazine) (MUHQOV; Näther *et al.*, 2002 ▸ and MUHQOV01; Zhang *et al.*, 2014 ▸) and (CuCl)_2_(2,6-di­methyl­pyrazine) (YEFPOR; Fan *et al.*, 2015*a* ▸). Moreover, we recently published two compounds with the composition (Cu*X*)_2_(2,6-di­methyl­pyrazine)_4_ (*X* = Cl, Br) that consist of dinuclear complexes (Näther, 2026*a* ▸). Some additional compounds are reported with copper(I) pseudohalides. These include Cu_2_(N_3_)_2_(2,6-di­methyl­pyrazine) (CUSFAZ; Fan *et al.*, 2015*b* ▸) and two different isomers of Cu_2_(CN)_2_(2,6-di­methyl­pyrazine) (GUZFIU; Näther, 2025 ▸ and SUYGAU (Chesnut *et al.*, 2001 ▸). Finally, CuNCS(2,6-di­methyl­pyrazine) is also reported (UYOYUG; Näther, 2026*b* ▸). There is also one mixed copper(I/II) pseudohalide compound with the composition [Cu_8_^I^Cu_2_^II^(CN)_4_-(NCS)_8_(2,6-di­methyl­pyrazine)_7_] (MEGLOA; Jess & Näther, 2006 ▸). Finally, it is noted that two different modifications with the composition CuBr_2_(2,6-di­methyl­pyrazine) are reported with divalent copper(II) cations in which the copper cations are linked into chains by bridging 2,6-di­methyl­pyrazine ligands (EWILAA; Ding *et al.*, 2021 ▸).

## 5. Synthesis and crystallization

Copper(I) iodide and 2,6-di­methyl­pyrazine were purchased from Sigma-Aldrich. To prepare the title compound, 0.5 mmol (95.2 mg) of copper(I) iodide and 0.25 mmol (27.0 mg of 2,6-di­methyl­pyrazine were heated in 2 ml of aceto­nitrile in a closed glass ampoule at 413 K for 1d. After annealing at 353 K for 2d, orange-colored crystals of (**I**) suitable for single crystal X-ray diffraction were obtained.

## 6. Refinement

Crystal data, data collection and structure refinement details are summarized in Table 3 ▸. The C—H hydrogen atoms were positioned with idealized geometry (methyl H atoms allowed to rotate but not to tip) and were refined isotropically with *U*_iso_(H) = 1.2 *U*_eq_(C) (1.5 for methyl H atoms).

## Supplementary Material

Crystal structure: contains datablock(s) I. DOI: 10.1107/S2056989026007358/hb8229sup1.cif

Structure factors: contains datablock(s) I. DOI: 10.1107/S2056989026007358/hb8229Isup2.hkl

CCDC reference: 2574159

Additional supporting information:  crystallographic information; 3D view; checkCIF report

## Figures and Tables

**Table 1:** Selected geometric parameters (Å, °)

Cu1—N1	2.018 (6)	Cu4—N11^ii^	2.001 (5)
Cu1—I1	2.5580 (9)	Cu4—I4	2.5340 (9)
Cu1—Cu2	2.5645 (12)	Cu4—I3	2.5754 (9)
Cu1—I2	2.5675 (9)	Cu5—N2^iii^	2.038 (6)
Cu2—I2	2.6187 (9)	Cu5—Cu6	2.5746 (11)
Cu2—I3	2.6487 (10)	Cu5—I6	2.6289 (9)
Cu2—I1	2.7765 (9)	Cu5—I5^iii^	2.6431 (10)
Cu3—N22	2.036 (5)	Cu5—I5	2.7467 (9)
Cu3—Cu4	2.5876 (11)	Cu6—N21	2.003 (5)
Cu3—I1^i^	2.6143 (9)	Cu6—I6	2.5480 (9)
Cu3—I4	2.6619 (9)	Cu6—I5	2.5693 (9)
Cu3—I3	2.7213 (9)		
			
N1—Cu1—I1	123.81 (14)	I4—Cu4—I3	122.43 (3)
N1—Cu1—I2	108.98 (14)	N21—Cu6—I6	116.24 (14)
I1—Cu1—I2	124.49 (4)	N21—Cu6—I5	122.07 (14)
N11^ii^—Cu4—I4	120.44 (13)	I6—Cu6—I5	121.69 (3)
N11^ii^—Cu4—I3	117.13 (13)		

Symmetry codes: (i) 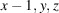; (ii) 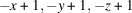; (iii) 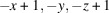.

**Table 2:** Hydrogen-bond geometry (Å, °)

*D*—H⋯*A*	*D*—H	H⋯*A*	*D*⋯*A*	*D*—H⋯*A*
C3—H3⋯I6^iv^	0.95	3.28	4.146 (7)	152
C5—H5*A*⋯I3	0.98	3.28	4.112 (7)	144
C5—H5*B*⋯I4^v^	0.98	3.11	4.030 (7)	157
C5—H5*C*⋯I2^v^	0.98	3.16	3.918 (7)	135
C6—H6*B*⋯I3^vi^	0.98	3.28	4.009 (7)	133
C6—H6*C*⋯I1^vii^	0.98	3.25	4.080 (7)	144
C12—H12⋯I3	0.95	3.29	3.888 (6)	123
C13—H13⋯I4^iv^	0.95	3.30	4.102 (7)	144
C15—H15*A*⋯I2^ii^	0.98	3.09	3.949 (7)	147
C15—H15*B*⋯I6^v^	0.98	3.23	4.205 (7)	173
C15—H15*C*⋯I4^ii^	0.98	3.32	4.089 (7)	137
C16—H16*C*⋯I6^viii^	0.98	3.20	4.106 (7)	155
C23—H23⋯I2	0.95	3.17	4.041 (7)	153
C25—H25*B*⋯I2^ix^	0.98	3.16	4.127 (7)	170
C25—H25*C*⋯I6^x^	0.98	3.19	4.027 (7)	144
C26—H26*A*⋯I3^vii^	0.98	3.26	4.204 (7)	163

Symmetry codes: (ii) 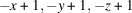; (iv) 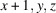; (v) 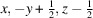; (vi) 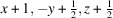; (vii) 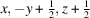; (viii) 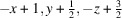; (ix) 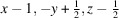; (x) 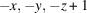.

**Table 3:** Experimental details

Crystal data	
Chemical formula	[Cu_2_I_2_(C_6_H_8_N_2_)]
*M*_r_	489.02
Crystal system, space group	Monoclinic, *P*2_1_/*c*
Temperature (K)	170
*a*, *b*, *c* (Å)	8.7105 (6), 26.5771 (12), 14.6153 (10)
β (°)	106.996 (8)
*V* (Å^3^)	3235.7 (4)
*Z*	12
Radiation type	Mo *K*α
μ (mm^−1^)	9.62
Crystal size (mm)	0.20 × 0.15 × 0.14

Data collection	
Diffractometer	Stoe IPDS-II
Absorption correction	Numerical (*X-RED* and *X-SHAPE*; Stoe, 2008 ▸)
*T*_min_, *T*_max_	0.237, 0.290
No. of measured, independent and observed [*I* > 2σ(*I*)] reflections	22517, 5539, 4665
*R*_int_	0.043
(sin θ/λ)_max_ (Å^−1^)	0.592

Refinement	
*R*[*F*^2^ > 2σ(*F*^2^)], *wR*(*F*^2^), *S*	0.031, 0.076, 1.03
No. of reflections	5539
No. of parameters	332
H-atom treatment	H-atom parameters constrained
Δρ_max_, Δρ_min_ (e Å^−3^)	2.80, −1.05

Computer programs: *X-AREA* (Stoe, 2008 ▸), *SHELXT* (Sheldrick, 2015*a* ▸), *SHELXL* (Sheldrick, 2015*b* ▸), *DIAMOND* (Brandenburg, 1999 ▸) *XP* in *SHELXTL-PC* (Sheldrick, 2008 ▸) and *publCIF* (Westrip, 2010 ▸).

## supplementary crystallographic information

### Poly[µ-2,6-dimethylpyrazine-µ3-iodido-µ2-iodido-dicopper(I)] Crystal data

**Table:**

[Cu_2_I_2_(C_6_H_8_N_2_)]	*F*(000) = 2664
*M**_r_* = 489.02	*D*_x_ = 3.012 Mg m^−^^3^
Monoclinic, *P*2_1_/*c*	Mo *K*α radiation, λ = 0.71073 Å
*a* = 8.7105 (6) Å	Cell parameters from 8000 reflections
*b* = 26.5771 (12) Å	θ = 8.8–22.3°
*c* = 14.6153 (10) Å	µ = 9.62 mm^−^^1^
β = 106.996 (8)°	*T* = 170 K
*V* = 3235.7 (4) Å^3^	Block, orange
*Z* = 12	0.20 × 0.15 × 0.14 mm

### Poly[µ-2,6-dimethylpyrazine-µ3-iodido-µ2-iodido-dicopper(I)] Data collection

**Table:**

Stoe IPDS-II diffractometer	4665 reflections with *I* > 2σ(*I*)
ω scans	*R*_int_ = 0.043
Absorption correction: numerical (X-Red and X-Shape; Stoe, 2008)	θ_max_ = 24.9°, θ_min_ = 2.1°
*T*_min_ = 0.237, *T*_max_ = 0.290	*h* = −10→10
22517 measured reflections	*k* = −30→30
5539 independent reflections	*l* = −17→17

### Poly[µ-2,6-dimethylpyrazine-µ3-iodido-µ2-iodido-dicopper(I)] Refinement

**Table:**

Refinement on *F*^2^	Hydrogen site location: inferred from neighbouring sites
Least-squares matrix: full	H-atom parameters constrained
*R*[*F*^2^ > 2σ(*F*^2^)] = 0.031	*w* = 1/[σ^2^(*F*_o_^2^) + (0.0444*P*)^2^ + 6.7618*P*] where *P* = (*F*_o_^2^ + 2*F*_c_^2^)/3
*wR*(*F*^2^) = 0.076	(Δ/σ)_max_ = 0.001
*S* = 1.03	Δρ_max_ = 2.80 e Å^−^^3^
5539 reflections	Δρ_min_ = −1.05 e Å^−^^3^
332 parameters	Extinction correction: SHELXL-2016/6 (Sheldrick 2015b), Fc^*^=kFc[1+0.001xFc^2^λ^3^/sin(2θ)]^-1/4^
0 restraints	Extinction coefficient: 0.00030 (4)

### Poly[µ-2,6-dimethylpyrazine-µ3-iodido-µ2-iodido-dicopper(I)] Special details

**Table:**

Geometry. All esds (except the esd in the dihedral angle between two l.s. planes) are estimated using the full covariance matrix. The cell esds are taken into account individually in the estimation of esds in distances, angles and torsion angles; correlations between esds in cell parameters are only used when they are defined by crystal symmetry. An approximate (isotropic) treatment of cell esds is used for estimating esds involving l.s. planes.

### Poly[µ-2,6-dimethylpyrazine-µ3-iodido-µ2-iodido-dicopper(I)] Fractional atomic coordinates and isotropic or equivalent isotropic displacement parameters (Å^2^)

**Table:**

	*x*	*y*	*z*	*U*_iso_*/*U*_eq_	
Cu1	0.73906 (11)	0.27611 (3)	0.46622 (6)	0.0249 (2)	
Cu2	0.58434 (10)	0.35949 (3)	0.44381 (6)	0.02364 (19)	
Cu3	0.07041 (10)	0.30994 (3)	0.42538 (6)	0.02246 (19)	
Cu4	0.22502 (10)	0.39156 (3)	0.49164 (6)	0.02336 (19)	
Cu5	0.37788 (10)	−0.02161 (3)	0.55017 (6)	0.0249 (2)	
Cu6	0.23067 (10)	0.05925 (3)	0.47579 (6)	0.02282 (19)	
I1	0.77362 (5)	0.33070 (2)	0.32945 (3)	0.01973 (11)	
I2	0.63723 (5)	0.30857 (2)	0.60343 (3)	0.01863 (11)	
I3	0.27865 (5)	0.35340 (2)	0.34192 (3)	0.01730 (11)	
I4	0.11827 (5)	0.33985 (2)	0.60538 (3)	0.02132 (12)	
I5	0.31851 (5)	0.00123 (2)	0.35969 (3)	0.01927 (11)	
I6	0.18317 (5)	0.02575 (2)	0.62860 (3)	0.02144 (12)	
N1	0.7177 (6)	0.2005 (2)	0.4594 (4)	0.0180 (11)	
C1	0.5891 (8)	0.1801 (2)	0.3920 (5)	0.0203 (14)	
C2	0.5645 (8)	0.1292 (3)	0.3898 (4)	0.0195 (14)	
H2	0.474983	0.115737	0.342180	0.023*	
N2	0.6611 (7)	0.0973 (2)	0.4521 (4)	0.0227 (12)	
C3	0.7866 (8)	0.1172 (2)	0.5182 (5)	0.0209 (14)	
H3	0.858018	0.095432	0.562198	0.025*	
C4	0.8164 (8)	0.1692 (2)	0.5246 (5)	0.0190 (13)	
C5	0.4770 (9)	0.2147 (3)	0.3240 (5)	0.0265 (15)	
H5A	0.442450	0.241355	0.359896	0.040*	
H5B	0.383073	0.195740	0.286594	0.040*	
H5C	0.531708	0.229688	0.280834	0.040*	
C6	0.9483 (8)	0.1913 (3)	0.6022 (5)	0.0263 (15)	
H6A	0.996618	0.219299	0.576667	0.039*	
H6B	1.030020	0.165576	0.628132	0.039*	
H6C	0.905812	0.203706	0.653081	0.039*	
N11	0.7301 (6)	0.5350 (2)	0.4847 (3)	0.0173 (11)	
C11	0.6051 (7)	0.5191 (2)	0.4093 (4)	0.0154 (13)	
C12	0.5656 (8)	0.4687 (2)	0.3974 (4)	0.0182 (13)	
H12	0.479831	0.458488	0.343853	0.022*	
N12	0.6461 (6)	0.4337 (2)	0.4599 (4)	0.0168 (11)	
C13	0.7717 (8)	0.4493 (2)	0.5320 (4)	0.0196 (14)	
H13	0.832846	0.425016	0.575237	0.024*	
C14	0.8154 (8)	0.4999 (2)	0.5455 (4)	0.0167 (13)	
C15	0.5146 (8)	0.5582 (2)	0.3406 (4)	0.0218 (14)	
H15A	0.461861	0.581372	0.373979	0.033*	
H15B	0.433508	0.541767	0.288158	0.033*	
H15C	0.589375	0.576967	0.314685	0.033*	
C16	0.9551 (8)	0.5162 (3)	0.6264 (5)	0.0248 (15)	
H16A	1.016126	0.541944	0.603928	0.037*	
H16B	1.024459	0.487204	0.650749	0.037*	
H16C	0.916449	0.530176	0.677690	0.037*	
N21	0.1948 (6)	0.1331 (2)	0.4513 (3)	0.0166 (11)	
C21	0.0673 (7)	0.1497 (2)	0.3786 (4)	0.0146 (12)	
C22	0.0358 (7)	0.2010 (2)	0.3670 (4)	0.0163 (13)	
H22	−0.052024	0.212005	0.315397	0.020*	
N22	0.1257 (6)	0.2354 (2)	0.4264 (4)	0.0187 (12)	
C23	0.2526 (8)	0.2187 (2)	0.4958 (4)	0.0182 (13)	
H23	0.320695	0.242434	0.536847	0.022*	
C24	0.2887 (7)	0.1674 (2)	0.5102 (4)	0.0157 (13)	
C25	−0.0361 (8)	0.1113 (3)	0.3147 (5)	0.0230 (14)	
H25A	0.028069	0.092376	0.281426	0.034*	
H25B	−0.124764	0.128173	0.267618	0.034*	
H25C	−0.079415	0.088146	0.353131	0.034*	
C26	0.4250 (8)	0.1498 (3)	0.5922 (5)	0.0246 (15)	
H26A	0.384915	0.141098	0.646254	0.037*	
H26B	0.505198	0.176595	0.611325	0.037*	
H26C	0.474338	0.120024	0.572819	0.037*	

### Poly[µ-2,6-dimethylpyrazine-µ3-iodido-µ2-iodido-dicopper(I)] Atomic displacement parameters (Å^2^)

**Table:**

	*U*^11^	*U*^22^	*U*^33^	*U*^12^	*U*^13^	*U*^23^
Cu1	0.0376 (5)	0.0144 (4)	0.0234 (4)	0.0062 (4)	0.0101 (4)	0.0028 (3)
Cu2	0.0271 (5)	0.0110 (4)	0.0293 (4)	−0.0007 (3)	0.0029 (4)	0.0004 (3)
Cu3	0.0245 (4)	0.0116 (4)	0.0288 (4)	0.0004 (3)	0.0038 (3)	0.0000 (3)
Cu4	0.0333 (5)	0.0138 (4)	0.0222 (4)	−0.0072 (3)	0.0069 (4)	−0.0019 (3)
Cu5	0.0304 (5)	0.0110 (4)	0.0313 (5)	0.0032 (3)	0.0059 (4)	0.0003 (3)
Cu6	0.0311 (5)	0.0138 (4)	0.0227 (4)	0.0070 (3)	0.0064 (4)	0.0048 (3)
I1	0.0151 (2)	0.0271 (3)	0.0173 (2)	0.00200 (16)	0.00537 (16)	0.00280 (16)
I2	0.0207 (2)	0.0190 (2)	0.0167 (2)	−0.00086 (16)	0.00640 (16)	−0.00025 (15)
I3	0.0153 (2)	0.0196 (2)	0.0170 (2)	−0.00201 (16)	0.00474 (16)	−0.00011 (15)
I4	0.0233 (2)	0.0241 (3)	0.0178 (2)	−0.00152 (17)	0.00786 (17)	0.00151 (16)
I5	0.0199 (2)	0.0202 (2)	0.0178 (2)	0.00073 (16)	0.00571 (17)	−0.00096 (16)
I6	0.0241 (2)	0.0218 (3)	0.0202 (2)	0.00382 (17)	0.00922 (18)	0.00317 (16)
N1	0.021 (3)	0.016 (3)	0.019 (3)	0.003 (2)	0.009 (2)	0.000 (2)
C1	0.022 (4)	0.016 (4)	0.024 (3)	0.004 (3)	0.009 (3)	−0.003 (3)
C2	0.015 (3)	0.028 (4)	0.014 (3)	0.002 (3)	0.003 (2)	−0.003 (3)
N2	0.024 (3)	0.014 (3)	0.032 (3)	0.001 (2)	0.010 (2)	0.001 (2)
C3	0.020 (3)	0.016 (4)	0.029 (3)	0.002 (3)	0.010 (3)	0.006 (3)
C4	0.022 (3)	0.016 (3)	0.025 (3)	0.002 (3)	0.015 (3)	0.005 (3)
C5	0.028 (4)	0.026 (4)	0.020 (3)	0.001 (3)	−0.001 (3)	0.004 (3)
C6	0.023 (4)	0.029 (4)	0.028 (4)	0.002 (3)	0.009 (3)	0.006 (3)
N11	0.019 (3)	0.021 (3)	0.013 (2)	−0.003 (2)	0.007 (2)	−0.003 (2)
C11	0.019 (3)	0.014 (3)	0.014 (3)	−0.001 (3)	0.007 (3)	−0.006 (2)
C12	0.017 (3)	0.016 (4)	0.020 (3)	0.000 (3)	0.002 (3)	0.000 (2)
N12	0.018 (3)	0.012 (3)	0.020 (3)	0.000 (2)	0.006 (2)	0.000 (2)
C13	0.021 (3)	0.017 (4)	0.022 (3)	0.001 (3)	0.007 (3)	0.002 (3)
C14	0.019 (3)	0.018 (4)	0.014 (3)	−0.001 (3)	0.006 (3)	−0.001 (2)
C15	0.031 (4)	0.017 (4)	0.014 (3)	0.001 (3)	0.002 (3)	0.002 (2)
C16	0.028 (4)	0.021 (4)	0.022 (3)	0.000 (3)	0.003 (3)	−0.001 (3)
N21	0.017 (3)	0.019 (3)	0.015 (3)	0.002 (2)	0.005 (2)	0.001 (2)
C21	0.013 (3)	0.015 (3)	0.017 (3)	−0.003 (2)	0.008 (2)	0.001 (2)
C22	0.013 (3)	0.014 (3)	0.022 (3)	0.001 (2)	0.006 (3)	0.007 (2)
N22	0.021 (3)	0.011 (3)	0.028 (3)	0.000 (2)	0.012 (2)	0.000 (2)
C23	0.020 (3)	0.014 (3)	0.023 (3)	0.000 (3)	0.009 (3)	−0.002 (3)
C24	0.016 (3)	0.015 (3)	0.018 (3)	0.003 (3)	0.009 (3)	−0.001 (2)
C25	0.029 (4)	0.015 (4)	0.022 (3)	−0.002 (3)	0.003 (3)	0.000 (3)
C26	0.020 (4)	0.025 (4)	0.024 (3)	0.001 (3)	−0.001 (3)	0.000 (3)

### Poly[µ-2,6-dimethylpyrazine-µ3-iodido-µ2-iodido-dicopper(I)] Geometric parameters (Å, º)

**Table:**

Cu1—N1	2.018 (6)	C5—H5C	0.9800
Cu1—I1	2.5580 (9)	C6—H6A	0.9800
Cu1—Cu2	2.5645 (12)	C6—H6B	0.9800
Cu1—I2	2.5675 (9)	C6—H6C	0.9800
Cu2—N12	2.040 (5)	N11—C14	1.350 (8)
Cu2—I2	2.6187 (9)	N11—C11	1.370 (8)
Cu2—I3	2.6487 (10)	C11—C12	1.381 (9)
Cu2—I1	2.7765 (9)	C11—C15	1.498 (9)
Cu3—N22	2.036 (5)	C12—N12	1.348 (8)
Cu3—Cu4	2.5876 (11)	C12—H12	0.9500
Cu3—I1^i^	2.6143 (9)	N12—C13	1.343 (9)
Cu3—I4	2.6619 (9)	C13—C14	1.398 (9)
Cu3—I3	2.7213 (9)	C13—H13	0.9500
Cu4—N11^ii^	2.001 (5)	C14—C16	1.491 (9)
Cu4—I4	2.5340 (9)	C15—H15A	0.9800
Cu4—I3	2.5754 (9)	C15—H15B	0.9800
Cu5—N2^iii^	2.038 (6)	C15—H15C	0.9800
Cu5—Cu6	2.5746 (11)	C16—H16A	0.9800
Cu5—I6	2.6289 (9)	C16—H16B	0.9800
Cu5—I5^iii^	2.6431 (10)	C16—H16C	0.9800
Cu5—I5	2.7467 (9)	N21—C24	1.352 (8)
Cu6—N21	2.003 (5)	N21—C21	1.367 (8)
Cu6—I6	2.5480 (9)	C21—C22	1.390 (9)
Cu6—I5	2.5693 (9)	C21—C25	1.495 (9)
N1—C4	1.363 (8)	C22—N22	1.345 (8)
N1—C1	1.369 (9)	C22—H22	0.9500
C1—C2	1.371 (10)	N22—C23	1.340 (9)
C1—C5	1.488 (9)	C23—C24	1.401 (9)
C2—N2	1.344 (9)	C23—H23	0.9500
C2—H2	0.9500	C24—C26	1.495 (9)
N2—C3	1.338 (9)	C25—H25A	0.9800
C3—C4	1.405 (10)	C25—H25B	0.9800
C3—H3	0.9500	C25—H25C	0.9800
C4—C6	1.479 (10)	C26—H26A	0.9800
C5—H5A	0.9800	C26—H26B	0.9800
C5—H5B	0.9800	C26—H26C	0.9800
			
N1—Cu1—I1	123.81 (14)	N1—C4—C3	119.1 (6)
N1—Cu1—Cu2	144.77 (16)	N1—C4—C6	118.9 (6)
I1—Cu1—Cu2	65.64 (3)	C3—C4—C6	121.9 (6)
N1—Cu1—I2	108.98 (14)	C1—C5—H5A	109.5
I1—Cu1—I2	124.49 (4)	C1—C5—H5B	109.5
Cu2—Cu1—I2	61.36 (3)	H5A—C5—H5B	109.5
N12—Cu2—Cu1	135.22 (15)	C1—C5—H5C	109.5
N12—Cu2—I2	115.16 (15)	H5A—C5—H5C	109.5
Cu1—Cu2—I2	59.37 (3)	H5B—C5—H5C	109.5
N12—Cu2—I3	108.17 (15)	C4—C6—H6A	109.5
Cu1—Cu2—I3	115.04 (4)	C4—C6—H6B	109.5
I2—Cu2—I3	110.82 (3)	H6A—C6—H6B	109.5
N12—Cu2—I1	98.80 (14)	C4—C6—H6C	109.5
Cu1—Cu2—I1	57.07 (3)	H6A—C6—H6C	109.5
I2—Cu2—I1	114.39 (3)	H6B—C6—H6C	109.5
I3—Cu2—I1	108.72 (3)	C14—N11—C11	118.2 (6)
N22—Cu3—Cu4	135.74 (16)	C14—N11—Cu4^ii^	121.1 (4)
N22—Cu3—I1^i^	113.81 (16)	C11—N11—Cu4^ii^	120.6 (4)
Cu4—Cu3—I1^i^	110.41 (4)	N11—C11—C12	120.7 (6)
N22—Cu3—I4	108.36 (15)	N11—C11—C15	117.7 (6)
Cu4—Cu3—I4	57.70 (3)	C12—C11—C15	121.6 (6)
I1^i^—Cu3—I4	108.16 (3)	N12—C12—C11	121.5 (6)
N22—Cu3—I3	103.21 (14)	N12—C12—H12	119.2
Cu4—Cu3—I3	57.97 (3)	C11—C12—H12	119.2
I1^i^—Cu3—I3	110.74 (3)	C13—N12—C12	117.5 (6)
I4—Cu3—I3	112.57 (3)	C13—N12—Cu2	121.0 (4)
N11^ii^—Cu4—I4	120.44 (13)	C12—N12—Cu2	121.4 (4)
N11^ii^—Cu4—I3	117.13 (13)	N12—C13—C14	122.2 (6)
I4—Cu4—I3	122.43 (3)	N12—C13—H13	118.9
N11^ii^—Cu4—Cu3	159.49 (16)	C14—C13—H13	118.9
I4—Cu4—Cu3	62.62 (3)	N11—C14—C13	119.8 (6)
I3—Cu4—Cu3	63.62 (3)	N11—C14—C16	119.1 (6)
N2^iii^—Cu5—Cu6	139.16 (17)	C13—C14—C16	121.1 (6)
N2^iii^—Cu5—I6	110.71 (15)	C11—C15—H15A	109.5
Cu6—Cu5—I6	58.63 (3)	C11—C15—H15B	109.5
N2^iii^—Cu5—I5^iii^	110.36 (17)	H15A—C15—H15B	109.5
Cu6—Cu5—I5^iii^	109.98 (4)	C11—C15—H15C	109.5
I6—Cu5—I5^iii^	111.32 (3)	H15A—C15—H15C	109.5
N2^iii^—Cu5—I5	102.74 (16)	H15B—C15—H15C	109.5
Cu6—Cu5—I5	57.63 (3)	C14—C16—H16A	109.5
I6—Cu5—I5	112.45 (3)	C14—C16—H16B	109.5
I5^iii^—Cu5—I5	108.93 (3)	H16A—C16—H16B	109.5
N21—Cu6—I6	116.24 (14)	C14—C16—H16C	109.5
N21—Cu6—I5	122.07 (14)	H16A—C16—H16C	109.5
I6—Cu6—I5	121.69 (3)	H16B—C16—H16C	109.5
N21—Cu6—Cu5	157.76 (16)	C24—N21—C21	118.7 (5)
I6—Cu6—Cu5	61.75 (3)	C24—N21—Cu6	120.9 (4)
I5—Cu6—Cu5	64.55 (3)	C21—N21—Cu6	120.3 (4)
Cu1—I1—Cu3^iv^	77.58 (3)	N21—C21—C22	119.9 (6)
Cu1—I1—Cu2	57.29 (3)	N21—C21—C25	118.0 (6)
Cu3^iv^—I1—Cu2	113.79 (3)	C22—C21—C25	122.1 (6)
Cu1—I2—Cu2	59.26 (3)	N22—C22—C21	122.1 (6)
Cu4—I3—Cu2	84.29 (3)	N22—C22—H22	118.9
Cu4—I3—Cu3	58.41 (3)	C21—C22—H22	118.9
Cu2—I3—Cu3	117.79 (3)	C23—N22—C22	117.2 (5)
Cu4—I4—Cu3	59.68 (3)	C23—N22—Cu3	118.0 (4)
Cu6—I5—Cu5^iii^	89.75 (3)	C22—N22—Cu3	124.5 (4)
Cu6—I5—Cu5	57.82 (3)	N22—C23—C24	122.5 (6)
Cu5^iii^—I5—Cu5	71.07 (3)	N22—C23—H23	118.8
Cu6—I6—Cu5	59.62 (3)	C24—C23—H23	118.8
C4—N1—C1	118.6 (6)	N21—C24—C23	119.5 (6)
C4—N1—Cu1	122.8 (5)	N21—C24—C26	119.3 (6)
C1—N1—Cu1	118.3 (4)	C23—C24—C26	121.1 (6)
N1—C1—C2	119.8 (6)	C21—C25—H25A	109.5
N1—C1—C5	118.4 (6)	C21—C25—H25B	109.5
C2—C1—C5	121.8 (6)	H25A—C25—H25B	109.5
N2—C2—C1	123.0 (6)	C21—C25—H25C	109.5
N2—C2—H2	118.5	H25A—C25—H25C	109.5
C1—C2—H2	118.5	H25B—C25—H25C	109.5
C3—N2—C2	117.2 (6)	C24—C26—H26A	109.5
C3—N2—Cu5^iii^	120.0 (5)	C24—C26—H26B	109.5
C2—N2—Cu5^iii^	122.9 (5)	H26A—C26—H26B	109.5
N2—C3—C4	122.3 (6)	C24—C26—H26C	109.5
N2—C3—H3	118.8	H26A—C26—H26C	109.5
C4—C3—H3	118.8	H26B—C26—H26C	109.5
			
C4—N1—C1—C2	−1.6 (8)	C12—N12—C13—C14	2.7 (9)
Cu1—N1—C1—C2	−175.3 (4)	Cu2—N12—C13—C14	−179.1 (4)
C4—N1—C1—C5	176.9 (5)	C11—N11—C14—C13	−2.1 (8)
Cu1—N1—C1—C5	3.3 (7)	Cu4^ii^—N11—C14—C13	174.1 (4)
N1—C1—C2—N2	0.5 (9)	C11—N11—C14—C16	178.1 (5)
C5—C1—C2—N2	−178.0 (6)	Cu4^ii^—N11—C14—C16	−5.7 (7)
C1—C2—N2—C3	−0.3 (9)	N12—C13—C14—N11	−0.1 (9)
C1—C2—N2—Cu5^iii^	179.1 (4)	N12—C13—C14—C16	179.7 (6)
C2—N2—C3—C4	1.3 (9)	C24—N21—C21—C22	−0.5 (8)
Cu5^iii^—N2—C3—C4	−178.1 (4)	Cu6—N21—C21—C22	174.8 (4)
C1—N1—C4—C3	2.5 (8)	C24—N21—C21—C25	−179.5 (5)
Cu1—N1—C4—C3	175.9 (4)	Cu6—N21—C21—C25	−4.2 (7)
C1—N1—C4—C6	−175.4 (5)	N21—C21—C22—N22	−1.1 (9)
Cu1—N1—C4—C6	−2.0 (7)	C25—C21—C22—N22	177.7 (5)
N2—C3—C4—N1	−2.4 (9)	C21—C22—N22—C23	2.8 (8)
N2—C3—C4—C6	175.4 (6)	C21—C22—N22—Cu3	−171.4 (4)
C14—N11—C11—C12	1.5 (8)	C22—N22—C23—C24	−2.8 (8)
Cu4^ii^—N11—C11—C12	−174.6 (4)	Cu3—N22—C23—C24	171.7 (4)
C14—N11—C11—C15	−178.1 (5)	C21—N21—C24—C23	0.5 (8)
Cu4^ii^—N11—C11—C15	5.8 (7)	Cu6—N21—C24—C23	−174.8 (4)
N11—C11—C12—N12	1.2 (9)	C21—N21—C24—C26	177.8 (5)
C15—C11—C12—N12	−179.2 (6)	Cu6—N21—C24—C26	2.5 (7)
C11—C12—N12—C13	−3.3 (8)	N22—C23—C24—N21	1.2 (9)
C11—C12—N12—Cu2	178.6 (4)	N22—C23—C24—C26	−176.0 (6)

Symmetry codes: (i) *x*−1, *y*, *z*; (ii) −*x*+1, −*y*+1, −*z*+1; (iii) −*x*+1, −*y*, −*z*+1; (iv) *x*+1, *y*, *z*.

### Poly[µ-2,6-dimethylpyrazine-µ3-iodido-µ2-iodido-dicopper(I)] Hydrogen-bond geometry (Å, º)

**Table:**

*D*—H···*A*	*D*—H	H···*A*	*D*···*A*	*D*—H···*A*
C3—H3···I6^iv^	0.95	3.28	4.146 (7)	152
C5—H5*A*···I3	0.98	3.28	4.112 (7)	144
C5—H5*B*···I4^v^	0.98	3.11	4.030 (7)	157
C5—H5*C*···I2^v^	0.98	3.16	3.918 (7)	135
C6—H6*B*···I3^vi^	0.98	3.28	4.009 (7)	133
C6—H6*C*···I1^vii^	0.98	3.25	4.080 (7)	144
C12—H12···I3	0.95	3.29	3.888 (6)	123
C13—H13···I4^iv^	0.95	3.30	4.102 (7)	144
C15—H15*A*···I2^ii^	0.98	3.09	3.949 (7)	147
C15—H15*B*···I6^v^	0.98	3.23	4.205 (7)	173
C15—H15*C*···I4^ii^	0.98	3.32	4.089 (7)	137
C16—H16*C*···I6^viii^	0.98	3.20	4.106 (7)	155
C23—H23···I2	0.95	3.17	4.041 (7)	153
C25—H25*B*···I2^ix^	0.98	3.16	4.127 (7)	170
C25—H25*C*···I6^x^	0.98	3.19	4.027 (7)	144
C26—H26*A*···I3^vii^	0.98	3.26	4.204 (7)	163

Symmetry codes: (ii) −*x*+1, −*y*+1, −*z*+1; (iv) *x*+1, *y*, *z*; (v) *x*, −*y*+1/2, *z*−1/2; (vi) *x*+1, −*y*+1/2, *z*+1/2; (vii) *x*, −*y*+1/2, *z*+1/2; (viii) −*x*+1, *y*+1/2, −*z*+3/2; (ix) *x*−1, −*y*+1/2, *z*−1/2; (x) −*x*, −*y*, −*z*+1.
